# Prediction of Antidepressant Treatment Outcome Using Event-Related Potential in Patients with Major Depressive Disorder

**DOI:** 10.3390/diagnostics10050276

**Published:** 2020-05-03

**Authors:** Hyun Seo Lee, Seung Yeon Baik, Yong-Wook Kim, Jeong-Youn Kim, Seung-Hwan Lee

**Affiliations:** 1 Clinical Emotion and Cognition Research Laboratory, Inje University, Goyang 50834, Korea; jslvfe77@gmail.com (H.S.L.); sybaik91@gmail.com (S.Y.B.); kim3863215@naver.com (Y.-W.K.); jeongyounk@gmail.com (J.-Y.K.); 2 Department of Biomedical Engineering, Hanyang University, Seoul 04763, Korea; 3 Department of Psychiatry, Inje University, Ilsan-Paik Hospital, Goyang 50834, Korea

**Keywords:** event-related potential (ERP), electroencephalogram (EEG), frontal alpha asymmetry (FAA), major depressive disorder, melancholic depression

## Abstract

(1) Background: Prediction of treatment outcome has been one of the core objectives in clinical research of patients with major depressive disorder (MDD). This study explored the possibility of event-related potential (ERP) markers to predict antidepressant treatment outcomes among MDD patients; (2) Methods: Fifty-two patients with MDD were recruited and evaluated through Hamilton depression (HAM-D), Hamilton anxiety rating scale (HAM-A), and CORE. Patients underwent a battery of ERP measures including frontal alpha symmetry (FAA) in the low alpha band (8–10 Hz), mismatch negativity (MMN), and loudness-dependent auditory evoked potentials (LDAEP); (3) Results: During the eight weeks of study, 61% of patients achieved remission, and 77% showed successful treatment responsiveness. Patients with low FAA in F5/F6 demonstrated a significantly higher remission/response ratio and better treatment responsiveness (*F* (2.560, 117.755) = 3.84, *p* = 0.016) compared to patients with high FAA. In addition, greater FAA in F7/F8 EEG channels was significantly associated with greater melancholia scores (*r* = 0.34, *p* = 0.018). Other ERP markers lacked any significant effect; (4) Conclusions: Our results suggested low FAA (i.e., greater left frontal activity) could reflect a good treatment response in MDD patients. These findings support that FAA could be a promising index in understanding both MDD and melancholic subtype.

## 1. Introduction

Major depressive disorder (MDD) is one of the most prevalent psychological disorders that presents a variety of chronic and recurring psychosocial hardships, which may even lead to one’s death [1,2]. According to previous studies, treatment outcomes among patients with MDD tend to show inconsistencies across research [3,4]. Depression presents a range of heterogenetic features in neurobiology, etiology, and symptomatology. For instance, it poses a clinical challenge when distinguishing the depressed phase of bipolar disorder from unipolar depression in a more acute way [5]. Further, studies related to suicide in MDD have suggested that suicidal patients differ from non-suicidal patients in many symptomatic structures, where depressed patients with suicidal ideation are characterized by a negative or pessimistic cognitive structure for the future (i.e., hopelessness) [6]. In their recent comprehensive review, Orsolini et al. [7] reported that suicide in MDD involves highly complicated mechanisms of both genetics and epigenetics. Melancholic and other atypical subtypes of depression also add to the heterogeneity within depression. One of the known factors of poor treatment outcome has been melancholic subtype [8,9]. Many findings have persistently argued that melancholic depression is a distinct subtype [10,11,12,13,14,15]. One clinical feature that is unique to melancholic patients when compared to non-melancholic patients is impaired cognitive function, as characterized by their low to no reactiveness to external stimulation and a decreased ability to regulate behavior as a function of reward [16,17,18,19,20]. Thus, the psychotherapeutic approach often proves less effective among melancholic patients, contributing to a treatment-resistant trait of melancholic depression [21,22]. Recent psychopharmacological findings on the role of the glutamatergic system at the basis of MDD pathophysiology have noted that esketamine may prove effective for treatment-resistant depression like melancholic depression [23,24].

In addition to significant implications of molecular alterations in MDD research, continued efforts towards evaluating biomarkers of MDD have included several event-related potential (ERP) measures, such as mismatch negativity (MMN), loudness-dependent auditory evoked potentials (LDAEP), and frontal alpha asymmetry (FAA) [25,26,27]. MMN is an ERP component elicited by randomly presented stimuli that differ in frequency or duration from the rest. It reflects an automatic detection of mismatch, or deviant sensory inputs, between the standard stimuli [28,29]. Studies have documented deficits in MMN among depressive patients [30,31], yet only a few studies have been done on its potential sensitivity for MDD treatment responses [32,33]. LDAEP, an auditory ERP that consists of monotonic increases of N1/P2 amplitude with varying tone intensities, has been studied as an indicator of serotonergic activity: a high LDAEP represents a low serotonergic level [34,35,36]. Nonetheless, clinical studies have shown inconsistencies in predicting treatment responsiveness using serotonergic agonists in patients with MDD [37,38].

Lastly, FAA has been frequently correlated with MDD severity [39,40,41,42], although with some degree of discrepancy across findings [43,44]. Studies have observed hemispheric lateralization due to reduced left to right frontal activity in alpha power and amplitude among depressive groups, including those who are at risk of depression [45,46,47], currently experiencing depression [48,49,50], or suffering from late-life and chronic depression [51,52,53]. In other words, increased left frontal activity has been linked to less depressive symptoms. As for FAA, the low alpha band (8–10 Hz) is thought to reflect a general attentional process such as selective attention to stimuli [54], and the high alpha band (10–12 Hz) is thought to represent a more complex and task-specific processing such as memory and reasoning [55]. Several FAA and depression studies have indicated low alpha band to function as a more sensitive indicator of clinical symptoms in depression, as low alpha frequency relates to a diverted attentional state [56,57,58]. Concerning asymmetry in specific channels, F5/F6 and F7/F8 have been considered notable phenotypes among at-risk, mild, and lifetime MDD patients [59,60,61,62,63,64]. In particular, asymmetry in F7/F8 has been studied in relation to dysfunctional anticipatory processing among melancholic individuals [65,66].

However, previous studies have suggested continuing heterogeneity and sometimes non-significant findings regarding FAA as a discriminator of MDD severity and patient treatment responsiveness. A recent meta-analytic review by Vinne et al. [44] posited that future evaluations of the predicting value of FAA would necessitate factoring in covariate influences such as sex, symptom severity, and comorbidity. The primary aim of the current exploratory study is to identify acute ERP biomarkers for MDD treatment outcomes while controlling for possible covariates. Furthermore, the present research specified the melancholic features among MDD patients to investigate the possible relation between ERP measures and current melancholia severity.

## 2. Materials and Methods

### 2.1. Participants

The present study was conducted by a retrospective chart review. A total of 52 patients with MDD between the ages of 18 and 65 (mean age: 45.87 ± 11.69) were consecutively recruited from early 2015 to late 2018. Participants were diagnosed according to the Diagnostic and Statistical Manual of Mental Disorders, Fifth Edition (APA), by board-certified psychiatrists. Patients with MDD were included in the study if they continued the antidepressant medication for at least eight weeks without discontinuation. All participants were either antidepressant-naive or did not take antidepressants for at least one month prior to participation. Prescribed antidepressant medications were restricted to vortioxetine (maximum dosage to 20 mg) or es-citalopram (maximum dosage to 20 mg), while other kinds of antidepressants were prohibited during the course of the study. The exclusion criteria were as follows: a history of psychotic symptoms, history of a substance use disorder, abnormal thyroid function test, neurological or internal diseases, pregnancy, and any history of treatment-resistance to antidepressant therapy or at high risk of suicidality. This study was approved by the Institutional Review Board at Inje University Ilsan Paik Hospital before the participation of the research (IRB no. 2018-012).

### 2.2. Psychological Assessments

To assess the severity of anxiety-related and depressive symptoms, the Hamilton Depression (HAM-D) [67] and Anxiety (HAM-A) [68] rating scales were used. HAM-D is composed of 17 items that measure different aspects of depression symptoms experienced over the past week on a scale of 0 to 4 (where 0 indicates the absence of the symptom and 4 indicates more acute signs of depression) while several questions extend up to 2 or 3. In general, the HAM-D score of 0 to 7 is considered within the normal range, whereas a score of 20 or higher falls in a clinical trial. HAM-D score was measured at four different time points in the treatment: baseline, 2nd week, 4th week, and 8th week. The scores measured at each week were used as a reference to define levels of depression severity and their change over the course of the study. The HAM-A measures the severity of both psychic (i.e., mental agitation and distress) and somatic (i.e., anxiety-related physical complaints) anxiety [69]. The scale consists of 14 items, and each item is scored on a scale of 0 (not present) to 4 (severe). A total score lower than 17 indicates mild severity, 18–24 mild to moderate severity, and 25–30 moderate to severe level of anxiety symptom severity. HAM-A score was measured at the baseline and the 8th week only.

For the evaluation of the melancholia subtype, the CORE [70] was used. CORE items include facial non-reactivity, facial apprehension, and delay in motor activity. The CORE measure evaluates a total of 18 observable features of melancholia on a four-point scale, ranging from 0 (absence of melancholia) to 3 (the highest severity of melancholia). A score equal to and higher than 8 was adopted in this study to diagnose melancholia [14]. CORE was measured only at the baseline of the study. Melancholia score was referred to as MEL in the analysis and data visualization.

### 2.3. EEG Recordings and Analysis

The EEG was obtained with Neuroscan SynAmps2 amplifier (Compumedics USA, El Paso, TX, USA) and with 64 Ag-AgCl electrodes mounted on a Quik-Cap using an extended 10–20 placement scheme. With an electrode cap on, participants were seated in a comfortable chair in a sound-attenuated room. The electrode was referenced at Cz and the ground electrode was placed on the forehead. A vertical electrooculogram (EOG) was recorded using bipolar electrodes: one was located above the right eye and one was located below. A horizontal EOG was recorded at the outer canthus of each eye. The impedance of the electrodes was maintained below 5 kΩ.

#### 2.3.1. Frontal Alpha Asymmetry (FAA)

The resting-state EEG data were recorded with a 0.1–100-Hz bandpass filter at a sampling rate of 1000 Hz, with 60 Hz noise removed using a notch filter. The data were preprocessed using Scan 4.3 software (Compumedics, El Paso, TX, USA). A trained individual with no information about the origin of the data manually removed gross movement artifacts. Ocular artifacts were removed through a mathematically programmed function in the preprocessing software [71]. The data were segmented into 2.048 s (2048 points) epochs, and the epochs with signals exceeding 100 μV on any of the 62 electrode sites were excluded from further analysis. In total, 30 epochs were prepared for each participant. FAA was defined as the lateral index by comparing the alpha frequency band percentages of the left and right hemispheres [63,64,72]. In the present study, the low alpha band (8–10 Hz) was considered a more acute indicator of clinical symptoms in depression [56,57,58]. A fast Fourier transformation was performed on the 62 electrodes to form the low alpha frequency band (8–10 Hz). This method includes measuring the difference between the two hemispheres using the equation: A = (P _left_ − P _right_)/(P _left_ + P _right_) × 100, where P _left_ and P _right_ each refers to the absolute powers of the corresponding frequency band in the applicable brain electrode. Hence, a positive value indicates greater alpha or reduced brain activity in the left hemisphere. A negative value indicates greater alpha or reduced brain activity in the right hemisphere. Channels F5/F6 and F7/F8 were chosen as the regions of interest based on previous findings [59,60,61,62,63,65,66].

#### 2.3.2. Loudness Dependent Auditory Evoked Potentials (LDAEP)

Auditory stimuli were generated by E-Prime software (Psychology Software Tools, Pittsburgh, PA, USA). Tones of 1000 Hz and 80-ms duration (10-ms rise and 10-ms fall, interstimulus interval randomized between 500 and 900 ms) were presented at five different sound pressure levels of intensities (60, 70, 80, 90, and 100 dB SPL) over MDR-D777 headphones (Sony, Tokyo, Japan) [73]. The collected data were then preprocessed using CURRY 7 (Compumedics, El Paso, TX, USA) and artifacts rejections were thoroughly inspected by a trained individual. The data were filtered using a 0.1–30 Hz bandpass filter and epoched from 100 ms pre-stimulus to 900 ms post-stimulus. The epochs were subtracted from the averaged pre-stimulus interval for baseline correction. Epochs with significant artifacts (amplitude exceeding ±75 μV) in any of the 62 electrode sites were rejected. Artifact-free epochs were then averaged across trials and participants for ERP analysis. They were calculated focusing on N1/P2 components for each participant: the N1 peak, which refers to the most negative peak between 50 and 200 ms from the stimulus, and the P2 peak, which refers to the most positive peak between 150 and 300 ms from the stimulus. The N1/P2 amplitudes were determined at the Cz electrode for the five different intensities. The peak-to-peak N1/P2 amplitudes were used to distinguish possible differences in participant’s responses to the varying tone intensities. The LDAEP was calculated as the slope of the linear regression.

#### 2.3.3. Mismatch Negativity (MMN)

The auditory stimuli were generated by E-Prime software (Psychology Software Tools, Pittsburgh, PA, USA). The stimuli consisted of sounds at 85 dB SPL and 1000 Hz: standard tones with a duration of 50 ms and deviant tones with a duration of 100 ms presented in a randomized order (probabilities: 10% and 90%, respectively). A total of 750 auditory stimuli were presented with an interstimulus interval of 500 ms. The rise and fall times were 10 ms, and the interstimulus interval was 1500 ms. The recorded data were preprocessed using CURRY 7 (Compumedics, El Paso, TX, USA) by a trained person and were filtered using a 0.1–30 Hz bandpass filter and epoched from 100 ms pre-stimulus to 600 ms post-stimulus. The epochs were subtracted from the averaged pre-stimulus interval value to correct for the baseline. After excluding artifacts with amplitude exceeding ± 75 μV in any site over 62 electrodes, the artifact-free epochs were averaged across trials and subjects for the following analysis. To calculate MMN wave values, we subtracted the standard ERP curves from the deviant curves. Given that greater amplitudes were shown in the area containing frontocentral electrodes, MMN amplitude was measured as the mean value between the time window of 130 and 280 ms at corresponding sites which were F3, Fz, F4, FC3, FCz, FC4, C3, Cz, and C4 [74]. The time window for the amplitudes was decided based on visual inspection of the grand-averaged waveforms at FCz.

### 2.4. Statistical Analysis

All indices, including FAA, LDAEP, and MMN, as well as MEL, were divided into low and high groups based on a median split. Remission was defined as a score of 7 or less in the HAM-D score at eight weeks. Successful treatment responsiveness was defined as a decrease of 50% or more in the HAM-D score at eight weeks compared to the baseline HAM-D score. In addition, the effect of group for each index on remission and the effect of group for each index on treatment response status was examined using chi-square analysis.

In order to assess the effects of all indices on remission and treatment response, we conducted repeated-measures ANOVA with the week (i.e., times points at baseline, 2nd, 4th, and 8th) as within variable and group (i.e., low and high groups for each index) as a between-group variable. Age, sex, HAM-D score at baseline, and medication types (i.e., vortioxetine and es-citalopram) were included as covariates. Mauchly’s test of sphericity was conducted to evaluate any violation of sphericity assumption. Greenhouse–Geisser correction was employed when a violation of the sphericity assumption was suggested. One-way ANCOVA analyses with age, sex, baseline HAM-D score, and medication types (i.e., vortioxetine and es-citalogram) as covariates were conducted to identify differences in the HAM-D score at each week of treatment between low and high groups.

As for an additional investigation of melancholia score possibly relating to other indices (i.e., FAA, LDAEP, and MMN), we conducted partial correlation analyses between the melancholia score and indices, controlling for age, sex, baseline HAM-D score, and medication types (i.e., vortioxetine and es-citalopram). *p*-value was set at 0.05. Statistical analyses and graphical analyses were carried out in IBM SPSS version 24 (IBM Corp., Armonk, NY, USA).

## 3. Results

### 3.1. Descriptive Statistics

After eight weeks of antidepressant treatment, 61% of MDD patients achieved remission and 77% of them exhibited a successful treatment response rate. A more detailed descriptive report on patient characteristics and a battery of psychological and neurophysiological measures given to participants is shown in Table 1.

### 3.2. Remission and Response Ratio

As for remission rate, a chi-square test demonstrated a significant group (low vs. high) difference in FAA F5/F6 (X^2^(1) = 5.20, *p* = 0.023). More specifically, patients who showed low FAA F5/F6 (i.e., greater left frontal brain activity) were more likely to be remitted than those who showed high FAA F5/F6 (i.e., reduced left frontal brain activity). Among those with low frontal alpha asymmetry (*n* = 26), 76.92% (20/26) achieved remission, whereas 23.08% (6/26) failed to achieve remission. As for treatment response rate, a significant group (low vs. high) difference in FAA F5/F6 was found (X^2^(1) = 3.90, *p* = 0.048). Patients who showed low FAA were more likely to show better responsiveness to the treatment than those who showed high FAA F5/F6. Among those with low frontal alpha asymmetry (*n* = 26), 88.46% (23/26) showed successful treatment responsiveness, whereas 11.54% (3/26) showed poor responsiveness.

**Figure 1:**
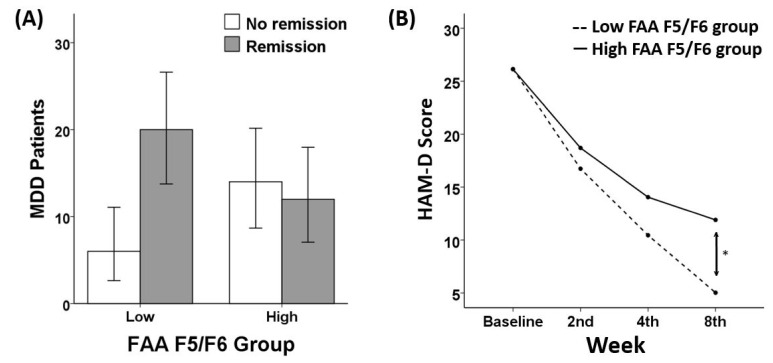
(**A**) Remission ratio by frontal alpha symmetry (FAA) F5/F6 groups (chi-square = 5.20, *p* = 0.023) and (**B**) treatment responsiveness in HAM-D score over the eight weeks by Low and High FAA F5/F6 groups (rANOVA: *F* (2.560, 117.755) = 3.84, *p* = 0.016). One-way ANCOVA showed a significant difference in HAM-D score between Low and High FAA group at 8th clinical benchmark, *p* = 0.009.

**Figure 2:**
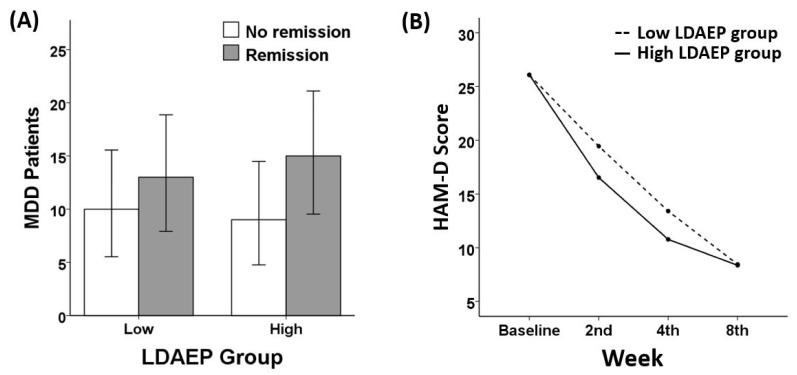
(**A**) Remission ratio by LDAEP groups (chi-square = 0.174, *p* = 0.676) and (**B**) treatment responsiveness in HAM-D score over the eight weeks by Low and High LDAEP groups (rANOVA: *F* (2.565, 105.182) = 1.183, *p* = 0.317).

**Figure 3:**
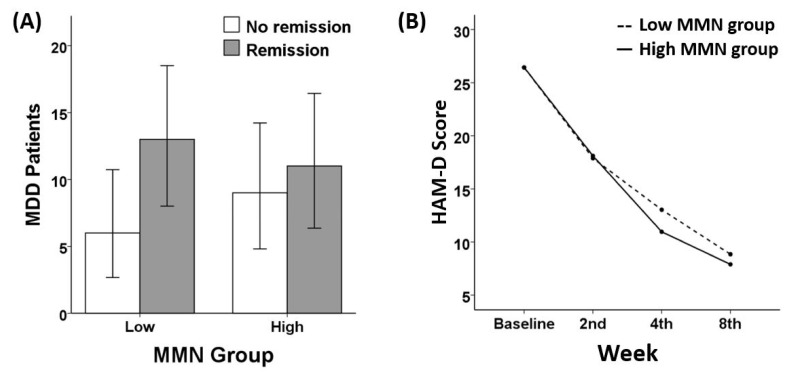
(**A**) Remission ratio by MMN groups (chi-square = 0.742, *p* = 0.389) and (**B**) treatment responsiveness in HAM-D score over the eight weeks by Low and High MMN groups (rANOVA: *F* (2.697, 88.992) = 0.373, *p* = 0.752).

**Figure 4:**
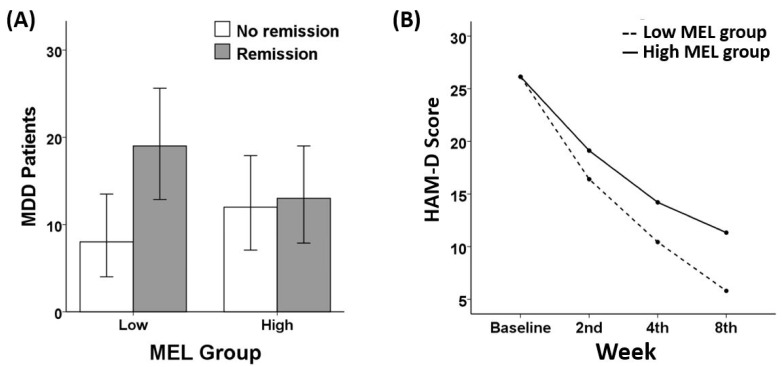
(**A**) Remission ratio by melancholic (MEL) groups (chi-square = 1.851, *p* = 0.174) and (**B**) treatment responsiveness in HAM-D score over the eight weeks by Low and High MEL groups (rANOVA: *F* (2.548, 117.185) = 2.474, *p* = 0.075).

However, a chi-square test revealed no significant group differences in FAA F7/F8 on remission status (X^2^(1) = 1.85, *p* = 0.174). There were no significant group differences regarding LDAEP (X^2^(1) = 0.174, *p* = 0.676), MMN (X^2^(1) = 0.742, *p* = 0.389), or MEL score (X^2^(1) = 1.851, *p* = 0.174). Similarly, no significant group differences in response rate were found regarding FAA F7/F8 (X^2^(1) = 0.257, *p* = 0.612), LDAEP (X^2^(1) = 0.181, *p* = 0.671), MMN (X^2^(1) = 0.242, *p* = 0.622), or MEL score (X^2^(1) = 0.657, *p* = 0.417). The chi-square analyses results of FAA F5/F6 (Figure 1), LDAEP (Figure 2), MMN (Figure 3), and melancholia score (Figure 4) are visualized in bar graphs.

### 3.3. Treatment Responsiveness (HAM-D)

**Figure 5:**
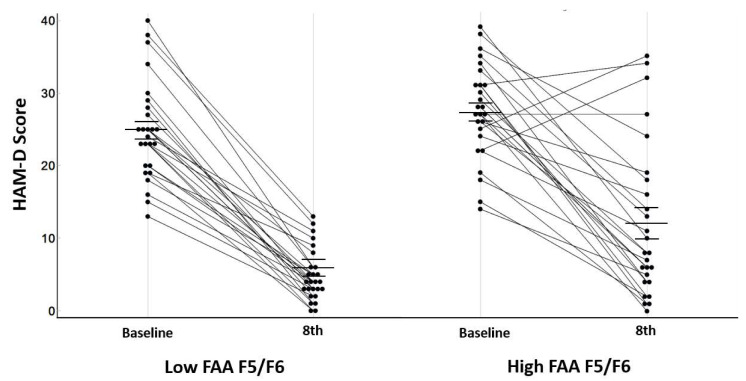
Parallel Coordinates of change in HAM-D scores from baseline to 8th week of treatment benchmark by Low and High FAA F5/F6 groups. Trend in Low FAA F5/F6 group is uniformly downward, whereas the trend in High FAA F5/F6 group is dispersed.

As for FAA, two separate repeated measures analyses for FAA in channels F5/F6 and for FAA in channels F7/F8 yielded different results. A significant interaction effect of week and FAA in channels F5/F6 group was shown (*F* (2.560, 117.755) = 3.84, *p* = 0.016). One-way ANCOVA analysis revealed that in the 8th week of treatment, a significant difference in the HAM-D score was found between the low FAA group (4.88 ± 3.60) and the high FAA group (12.04 ± 10.62) with *p* = 0.009. In contrast, regarding the FAA in channels F7/F8 group, no significant interaction effect was found (*F* (2.464, 113.347) = 1.00, *p* = 0.383). There were no significant interactions between week and other indices: LDAEP (*F* (2.565, 105.182) = 1.183, *p* = 0.317), MMN (*F* (2.697, 88.992) = 0.373, *p* = 0.752), and melancholia score (*F* (2.548, 117.185) = 2.474, *p* = 0.075). In addition, there were no significant group differences in the HAM-D score at each week of treatment as for LDAEP, MMN, and melancholia score. Among all indices of ERP, FAA emerged as the only variable showing a robust sensitivity to the remission status. The repeated measures results of FAA F5/F6 (Figure 1), LDAEP (Figure 2), MMN (Figure 3), and melancholia score (Figure 4) are shown in line charts. Additionally, the change in HAM-D score from the baseline to the 8th week of treatment by low and high FAA F5/F6 groups is visualized through the parallel coordinate plot (Figure 5).

### 3.4. Melancholia Score Correlation Analyses

**Figure 6:**
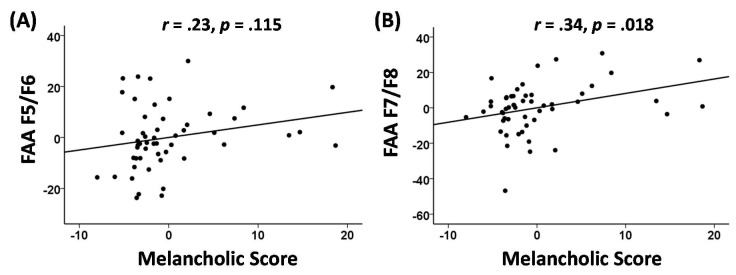
Partial correlation (age, sex, HAM-D at baseline, and medication types as covariates) plots of the positive relation between (**A**) FAA F5/F6 and melancholia score (*r* = 0.23, *p* = 0.115), and (**B**) FAA F7/F8 and melancholia score (*r* = 0.34, *p* = 0.018).

Partial correlation analyses yielded that only FAA in channels F7/F8 showed a significant correlation with the melancholia score. Even after controlling for age, sex, HAMD-score at baseline, and medication types, FAA in channels F7/F8 and the melancholia score revealed a significant positive relationship, with *r* = 0.34 with *p* = 0.018. As for FAA in channels F5/F6, the partial correlation analysis yielded a non-significant result (*r* = 0.23, *p* = 0.115). Melancholia score showed no significant correlation with LDAEP (*r* = −0.17, *p* = 0.253) or MMN (*r* = 0.18, *p* = 0.294). Partial correlation plots of the results are presented in Figure 6.

## 4. Discussion

The present study aimed to identify and evaluate possible EEG indices such as FAA, MMN, and LDAEP, along with the MEL score, for the treatment outcome of MDD patients. Our major findings are as follows. First, after 8-week long treatment of appropriate antidepressant medication, 61% of patients achieved remission, and 77% showed a successful treatment response. Second, MDD patients with low scores of FAA in channels F5/F6 returned a significantly higher remission/response ratio and better treatment responsiveness compared to those with high scores of FAA in channels F5/F6. Lastly, FAA in channels F7/F8 showed a significant positive correlation with melancholic depression scores.

First, among recruited MDD patients, a high rate of remission and successful treatment response was observed. Of all patients, 61% achieved remission, and 77% exhibited a successful treatment response during the 8-week course of the study. According to a previous study, more than 40% of patients with MDD failed to achieve remission following multiple trials of antidepressant medications [75]. The general range of remission rate falls between 37.5% and 67%, whereas the treatment response rate hovers around 62% [76,77,78]. Given this, the patients included in this study showed considerably higher than average remission and treatment response rates without any treatment-resistant problems.

Second, MDD patients with low scores of FAA in channels F5/F6 showed a significantly higher remission/response ratio and better treatment responsiveness compared to those with high scores of FAA in channels F5/F6. This finding is in line with pre-existing findings, where reduced left-hemispheric activation is thought to reflect brain physiology linked with depression and anxiety, while increased activation suggests otherwise [48,50,53,79]. FAA has been understood as a measure of an individual’s emotional state of endorsing approaching or withdrawing behavior [80,81,82]. Individuals with greater resting left frontal activity (i.e., low FAA) tend to select more pleasant stimuli in a later judgment task compared with subjects with greater resting right-sided frontal activity (i.e., high FAA). That is, relatively greater left than right frontal activity characterizes approach-oriented traits and states. On the other hand, greater right than left frontal activity is considered to demonstrate withdrawal-related traits and states. Several findings have consistently reported that the MDD group showed distinctly greater alpha in the left-frontal region (i.e., high FAA), which represents reduced left-frontal activity, in comparison to controls [50,60,79].

In the present study, MDD patients with relatively low left frontal activity (i.e., high FAA) are more likely to be associated with poor outcomes in terms of remission status. In contrast, those with high left frontal activity (i.e., low FAA) are more likely to be associated with better treatment outcomes. Given the approach-withdrawal model, the tendency of general avoidance due to reactiveness to negative valences such as fear and sorrow is more prevalent and persistent among patients with lower left frontal activity (i.e., high FAA) [83]. They are more prone to act upon withdrawal-related motivations. This tendency may lead to patient’s disengagement from the treatment and counseling. In contrast, as for patients with higher left frontal activity (i.e., low FAA), their tendency of general proactiveness may contribute to their engagement in coping and therapeutic actions suggested by surrounding support networks and clinicians. They could show relatively better outcomes when in treatment and under medications.

Third, greater FAA in channels F7/F8 was associated with greater melancholia scores, after controlling for age, sex, HAM-D score at baseline, and medication types. In the present study, frontal alpha asymmetry in F5/F6 was linked to patient’s treatment outcomes, and frontal alpha asymmetry in F7/F8 was associated with greater melancholia severity. These associations could imply a region-specific difference regarding patients with a varying range of melancholic features: patients with more severe melancholic features tended to show greater frontal alpha asymmetry in the F7/F8 area in comparison to patients with less severe melancholic features. The F7/F8 pair represents the dorsolateral prefrontal cortex (DLPFC), a region primarily associated with executive cognitive control, including cognitive flexibility [84,85], regulation of emotional processing [86,87], and working memory [88]. Clinical studies in psychiatry have identified anticipatory reward processing as one of the primary functions of the DLPFC [89,90,91,92]. Both functional and structural neuroimaging studies involving melancholic subtypes have pointed to an escalated hemispheric imbalance in the DLPFC in comparison to the non-melancholic depressive group [93,94,95,96]. Seemingly, patients with melancholic features are prone to suffer from DLPFC-related functions. Depressive patients with melancholic features tend to present a broader range of prolonged and pronounced cognitive impairments that include dysfunctional anticipatory systems [65,96,97,98] and hampered cognitive control in information processing [20,99,100,101]. The current finding strengthens the connection of melancholic features to the DLPFC and its primary functions; heightened hemispheric asymmetry or imbalance in the DLPFC region may indicate exacerbated cognitive deterioration. In short, the result supports the notion that cognitive impairment may play a role as one of the key melancholic phenotypes.

Additionally, it is worthy of note that the correlation between FAA and melancholia severity remained significant regardless of patients’ unipolar depression severity. This specifies a notable heterogeneity within the MDD group. The fact that F7/F8, and not F5/F6, related to melancholic severity seems to indicate that FAA measure could be used in differentiating a specific subtype of MDD. This provides evidence with regards to a distinct neurological understanding of the melancholic subtype in terms not only of its clinical characteristics and but also of how treatment responsiveness may vary from non-melancholic depressive groups. Future studies may address ways to further refine phenotypic distinctions for subtyping MDD.

Psychopathological heterogeneity exists in depression and possible subtypes may complicate this issue. Thus, symptomatological heterogeneity also lies both within and between different depression subtypes, which adds more clinical complications to the diagnosis and treatment. Empirical studies have utilized neurobiological methods as part of continuing works to improve the diagnostic and treatment quality of various depression subtypes. The findings in the present study provide further evidence that FAA may be a reliable ERP biomarker for MDD treatment outcomes in terms of remission status. In addition, this correlation finding indicates that FAA represents melancholic tendency of patients with MDD. Taken together, the present study states a possible efficacy of frontal alpha asymmetry index in detecting the MDD treatment responsiveness and the distinct melancholia severity within MDD groups.

Despite the significance of the findings, some limitations should be addressed. First, the number of patients was small, and the antidepressant effects were tested on this small size of the cohort. Future studies may benefit from including a larger sample. A second limitation is that only a small number of male patients with MDD were included in the study. Despite the fact that females tend to report more depressive symptoms than males [102] and that we controlled for the sex variable in the current study, samples with less gender bias are generally preferred. Third, this study was conducted by a retrospective chart review and the prescribed medications were restricted to two drugs (i.e., vortioxetine and es-citapalogram). This may suggest the sample vulnerability to selection bias. Lastly, this study has collected melancholia scores only at the baseline. A series of follow up assessments of melancholia severity (e.g., from baseline to 8th week of treatment) would provide better observation of possible changes in melancholia severity related to frontal alpha asymmetry over time as well as the possible dynamicity and variants within melancholic features.

## 5. Conclusions

The findings demonstrated that low FAA, or heightened left frontal activity, could indicate a better treatment outcome in terms of remission status among patients with MDD. In addition, FAA is likely to represent a melancholic tendency in patients with MDD. In sum, our results suggest that FAA could be a reliable biomarker to predict remission in the treatment of patients with MDD.

## Figures and Tables

**Table 1:** Descriptive statistics of remission status, treatment response, psychological measure, and neurophysiological measure data of participants.

Variable	Patient (*n* = 52)
**REMISSION STATUS**	N (%)
Yes	32 (61.54)
No	20 (38.46)
**TREATMENT RESPONSE**	
Successful	40 (76.92)
Unsuccessful	12 (23.08)
**PSYCHOLOGICAL MEASURE**	
Age (year)	45.87 ± 11.69
Sex	
Male	4 (7.69)
Female	48 (92.31)
Education (year)	13.42 ± 2.89
HAM-D (baseline)	26.13 ± 6.80
HAM-A (baseline)	25.13 ± 6.02
CORE (baseline)	4.35 ± 5.98
**NEUROPHYSIOLOGICAL MEASURE**	
LDAEP (μV/10 dB)	1.55 ± 2.85
MMN (μV)	−2.97 ± 1.34
Alpha asymmetry index of F5/F6	
Low alpha band	0.03 ± 14.13
High alpha band	−1.29 ± 13.16
Alpha band	−1.05 ± 12.73
Alpha asymmetry index of F7/F8	
Low alpha band	−1.73 ± 15.81
High alpha band	−2.96 ± 13.56
Alpha band	−2.80 ± 14.74

The data are shown as mean ± standard deviation (SD). HAM-D, Hamilton Rating Scale for Depression; HAM-A, Hamilton Rating Scale for Anxiety; LDAEP(dB), Loudness Dependence of Auditory Evoked Potentials; MMN(μV), Mismatch Negativity. Only the low alpha bands were included in the analysis.
